# The association between hyperkyphosis and physical function in geriatric outpatients with frailty

**DOI:** 10.1007/s11657-025-01619-z

**Published:** 2026-01-08

**Authors:** Marije C. Koelé, Nathalie van der Velde, Jos P.C.M. van Campen, Saskia A.E. van Haelst, Hanna C. Willems

**Affiliations:** 1 Amsterdam UMC location University of Amsterdam, Department of Internal Medicine, Division of Geriatrics, Meibergdreef 9, Amsterdam, 1105 AZ the Netherlands; 2 Department of Geriatrics, OLVG hospital, Jan Tooropstraat 164, Amsterdam, 1061 AE the Netherlands; 3 Amsterdam Public Health, Aging & Later Life, Amsterdam, the Netherlands; 4 Amsterdam Movement Sciences, Ageing & Vitality, Amsterdam, the Netherlands

**Keywords:** Kyphosis, Physical Function, Healthy aging

## Abstract

**Summary:**

Currently, it is unknown whether hyperkyphosis is associated with lower physical function in older persons with frailty, who have a high risk of functional decline. Hyperkyphosis was highly prevalent and independently associated with lower physical function. Early detection and treatment of hyperkyphosis may contribute to the preservation of physical function.

**Purpose:**

To investigate the association between hyperkyphosis and physical function in older persons with frailty, who have a high risk of functional decline

**Methods:**

Hyperkyphosis was defined as a Cobb angle ≥ 50°, ≥ 3.4 cm of blocks or an occiput-to-wall distance of ≥ 5.0 cm. The association with physical function (Timed Up and Go Test, Short Physical Performance Battery, Berg Balance Scale, and hand grip strength) was assessed through multifactorial regression analyses.

**Results:**

Hyperkyphosis was highly prevalent in the cohort (*n* = 337, mean age 80.0 ± 7.9 years) with a prevalence ranging from 43 to 84%, depending on which measurement method was used. Hyperkyphosis was independently associated with a longer Timed Up and Go time and a lower Berg Balance Scale score, only when kyphosis was measured with the blocks method (Timed Up and Go: adjusted OR 3.86, 95% CI 1.50–9.91; Berg Balance Scale: adjusted OR 9.42, 95% CI 2.27–39.12). Hyperkyphosis was not associated with Short Physical Function Battery or hand grip strength.

**Conclusion:**

Hyperkyphosis was highly prevalent and independently associated with the Timed Up and Go test and Berg Balance Scale, even in this population of geriatric outpatients with frailty and multimorbidity, thereby more at risk for functional decline. Early detection and treatment of hyperkyphosis may contribute to the preservation of physical function.

## Introduction

Hyperkyphosis is an excessive curvature of the thoracic spine. The prevalence rises with age to 15–40% among community-dwelling older persons [1, 2]. Several studies have investigated the association between hyperkyphosis and physical function [1, 3–14]. A recent meta-analysis of Roghani et al. confirmed a statistically significant association between hyperkyphosis and lower physical function in older females [10]. In older persons with frailty, an even higher hyperkyphosis prevalence of 55% has been reported [15]. These patients have a lower level of physical function and more chronic diseases [16]. Consequently, they have a higher risk of losing muscle strength and more functional limitations, ultimately leading to functional dependence [17, 18]. Older persons highly value functional independence [19, 20], often outweighing other aspects of well-being. Additionally, functional dependence causes high costs for society as well as for the individual [21]. Functional independence in later life can be achieved by maintaining physical function [22, 23]. Therefore, it is important to know all determinants contributing to physical function. However, it is unknown if hyperkyphosis contributes to functional decline in persons at high risk of functional deterioration, for instance, because of multimorbidity. An association that is clearly present in the general population may disappear in older persons with frailty and multimorbidity due to competing risks. A well-known example of competing risks in older persons is the cholesterol paradox. Elevated LDL cholesterol levels are associated with a higher mortality risk in the general population. Yet in older persons with frailty, lower cholesterol levels are associated with higher mortality because they may not live long enough to develop cardiovascular disease, and lower levels of cholesterol may be seen as a marker of underlying illness [24]. Therefore, it is important to investigate the association between hyperkyphosis and physical function in older persons with frailty. Because of the higher hyperkyphosis prevalence and higher risk of functional decline, it may be hypothesized that hyperkyphosis is more relevant in persons with frailty and multimorbidity. Alternatively, hyperkyphosis could be too minor to add to this risk due to all other causes of functional decline in this population. In order to maintain physical function in those most at risk of physical decline, more studies are warranted in persons with frailty.

**Fig. 1:**
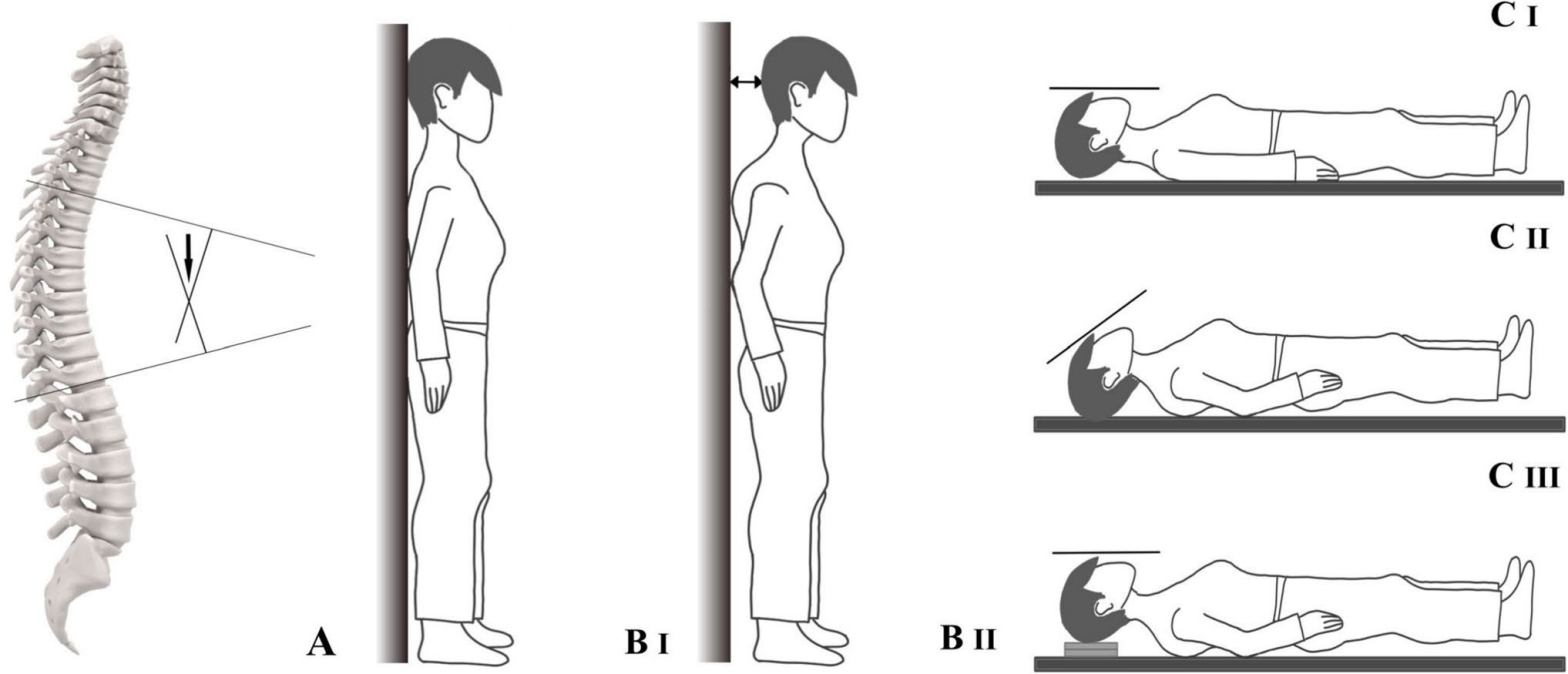
Three kyphosis measurements: **A** Cobb angle, **B** Occiput-to-wall distance (OWD), **C** blocks method

Additionally, because of the large variety of kyphosis measurement methods used, study results are difficult to compare. This is reflected in the large range of hyperkyphosis prevalences reported in the literature. The measurement methods currently in use in research vary substantially depending on the position of the person during the measurement and the part of the spine being measured. The Cobb angle (Fig. 1A) is the current gold standard measurement and is commonly measured in a standing position on the thoracic part of the spine. The occiput-to-wall distance (OWD, Fig. 1B) is also a measurement in a standing position and takes into account the whole spine. During the blocks method (Fig. 1C), the person is in a supine position.

The kyphosis angle is influenced by many factors, such as the anatomy of the vertebra and intervertebral discs, ligaments, and back extensor muscle strength [25]. As the influence of these factors on the kyphosis angle may differ per position, the type of kyphosis measurement method may affect the kyphosis angle. However, to the best of our knowledge, only three studies on clinically relevant outcomes have used multiple kyphosis measurement methods concurrently. These studies concern fall risk and vertebral fractures, and not physical function [15, 26, 27].

This study aimed to investigate the association between hyperkyphosis, quantified with two to three measurement methods concurrently, and physical function in older persons with frailty. We hypothesized that hyperkyphosis, despite other potential factors influencing the functional status, would be associated with physical function.

## Methods

### Participants

Persons referred to the geriatric outpatient clinic of the MC Slotervaart and the Amsterdam University Medical Centre were consecutively recruited on the days the researcher was present between December 2016 and March 2018 if they were able to participate in at least two of the three kyphosis measurements and one of the physical function tests. They were referred to the outpatient clinic and underwent a comprehensive geriatric assessment because they are frail and experience various health problems, such as memory complaints and falls. Lateral radiographs of the thorax and blood sampling were part of the comprehensive geriatric assessment. All procedures performed in the study were in accordance with the ethical standards of the institutional research committee and with the 1964 Helsinki Declaration and its later amendments. All participants gave their written informed consent. The medical ethics committee of the Amsterdam University Medical Centre approved the study and judged it not subject to the Medical Research (Human Subjects) Act.

### Kyphosis measurements

#### Cobb angle [28]

The Cobb angle (Fig. 1A) is commonly measured between the superior endplate of the fourth (T4) and the inferior endplate of the twelfth thoracic vertebra (T12) [28, 29]. The angle between the fourth and twelfth vertebra was assessed on lateral radiographs, taken in the upright position. Hyperkyphosis was defined as a Cobb angle ≥ 50°. If either vertebra T4 or T12 was not visible, the adjacent visible vertebra was used. Two researchers (MK, SH) assessed the Cobb angle of all participants independently. If the two measurements differed by less than 10° [30], the mean value was calculated. A third researcher (HW) remeasured the Cobb angle if the difference was 10° or more [30] or if the Cobb angle was judged to be more than 50° by one researcher and less than 50° by the other researcher. Based on consensus, HW and MK decided after remeasurement what the final value was of the Cobb angle.

#### OWD [31]

The OWD (Fig. 1B) was measured by asking participants to stand shoeless with their backs against the wall, with their heels as close as possible to the wall and their knees as extended as far as possible while relaxing their shoulders and neck. The distance between the occiput and the wall was measured with a ruler. Hyperkyphosis was defined as ≥ 5.0 cm [15].

#### Blocks method [2]

Persons with hyperkyphosis must hyperextend their neck to be able to lay down on a flat surface. While in a supine position, the distance from the occiput to the research table was measured by putting blocks underneath the head of the participant until the neck was neither hyperextended nor flexed (Fig. 1C). Kado et al. found an association with physical function for 2 blocks (3.4 cm) or more. The association with 1 block (1.7 cm) was non-significant in this study. In order to be able to compare our results to previous studies, hyperkyphosis was defined as ≥ 3.4 cm of blocks needed to achieve this neutral position of the head [3].

### Physical function

Physical function was assessed through the following tests: Timed-Up and Go test (TUG)[32], Short Physical Function Battery (SPPB)[33], Berg Balance Scale (BBS)[34], and hand grip strength (HGS). The set of physical function tests in clinical practice differed between the two study centers. Grip strength was measured in both study centers, the SPPB and BBS were only conducted in the AMC hospital, and the TUG was mainly executed by participants included in the AMC hospital and in a small subgroup in the MC Slotervaart. Thus, the TUG, SPPB, and BBS were only performed in a subgroup of the total cohort.

The TUG is widely used in daily clinical practice to detect mobility impairments and estimate fall risk. During the TUG, the participant is asked to stand up from a chair with a standardized seat height of 48 cm, walk 3 m, turn, and return to a seated position in the chair [32]. Persons were not allowed to use their arms to push up and were allowed to use their walking aid only if they were not able to walk. Based on previous literature, a TUG time of more than 15 s was chosen to distinguish between normal and decreased physical function[35].

A balance test, chair stand test, and gait speed together form the SPPB. Based on the classification of Guralnik et al., a score of 9–12 points was considered normal, and a score of eight points or less indicated limitations in physical function [33]. The balance test comprises three positions. The time participants can maintain that position (0–10 s) was registered. Firstly, participants were asked to stand with their feet as close as possible side by side. Secondly, the participants were asked to stand 10 s in a semi-tandem stand with one foot half beside the other foot. Thirdly, they performed the tandem stand, which means that one foot is placed right before the other foot. During the chair stand test, the participant’s ability to stand up five times from and sit down from a chair with a standard seat height of 48 cm was established. If possible, participants were encouraged to cross their arms at the chest. Ng et al. demonstrated that foot placement, but not arm position, affects the time required to complete the five chair stands [36]. Thus, although the most common protocol requires the participants to cross their arms at the chest, participants were allowed to use their hands while standing up only if they were not able to perform the test with their arms in that position. The time to complete these five chair stands was recorded in seconds. Gait speed (m/s) at a usual pace was measured over a distance of 4 m. Only if needed, they were allowed to use their walking aid. The TUG and SPPB were guided by the researchers (MK, SH) or the clinical doctors. They were trained by the researchers to ensure the reliability and validity of the functional tests. They had a written manual available at the outpatient clinic.

The Berg Balance Scale consists of 14 items with 0–4 points per item, through which balance during several positions and transfers is assessed. A score of 45 points or below is associated with a higher fall risk and is used to define normal versus decreased balance [34]. The BBS was guided by the physiotherapist of the department.

Hand grip strength was measured using the JAMAR Hydraulic Hand Dynamometer (Sammons Preston Rolyan, USA) in the MC Slotervaart and the Takei 5401 Hand Grip Dynamometer (Takei Equipment Industrial) in the Amsterdam UMC. Participants held the dynamometer in the hand with their arm dangling straight while standing, with the elbow and wrist in a neutral position, and pressed the dynamometer as much as possible. When unable to stand, the measurement was performed in a sitting position with the arm dangling straight. The maximal result of four trials, namely two per hand with a pause between measurements, was recorded by the researchers (MK, SH) [37]. The maximal result of several trials may be more representative of physical function than the mean value in this population with comorbidities affecting one side of the body more than the other side, such as rheumatic diseases, Parkinson’s disease, and unilateral trauma. Linear regression analysis was performed, so there was no cut-off score to define lower physical function.

### Covariables

All covariables were assessed on the day of the visit to the outpatient clinic. Age, sex, alcohol use, smoking, information on the use of a walking aid, comorbidity, and current medication use were derived from the medical record. Functional status was assessed by the Katz-ADL and Katz-IADL. A participant was described as physically inactive if he or she sits for 4 h or more per day, goes for a walk less than once a month, and does not cycle or jog [38]. Cognitive function was examined by the Mini-Mental State Examination (MMSE) and symptoms of a depressive disorder by the Geriatric Depression Scale (GDS). As blood sampling was part of the Comprehensive Geriatric Assessment, the 25-hydroxy-vitamin-D concentration was also available in the medical record.

### Statistical analysis

Baseline characteristics were determined for the overall group and for participants with hyperkyphosis versus participants with a normal kyphosis angle. Differences between groups were tested using a *t*-test for continuous variables, a chi-square test for categorical variables, and a Mann–Whitney U test if variables were non-normally distributed.

Binary logistic regression was performed for the TUG, SPPB, and BBS, based on the cut-off scores explained above (i.e., TUG ≥ 15 s, SPPB ≤ 9 points, and BBS ≤ 45 points). Linear regression analysis was performed for hand grip strength. The regression models were adjusted for potential confounders, i.e., age, BMI, current or former smoking, alcohol use, fracture after the age of 50, and vitamin D level. Through backward selection, variables were added to the model. A priori, we decided to stratify for sex in all analyses, as overlapping results between the women with the best function and men with the worst function may lead to a type II error. The level of significance was set at *α* = 0.05. Statistical analyses were performed with SPSS software(Statistical Package for the Social Sciences) version 21.0.

## Results

**Fig. 2:**
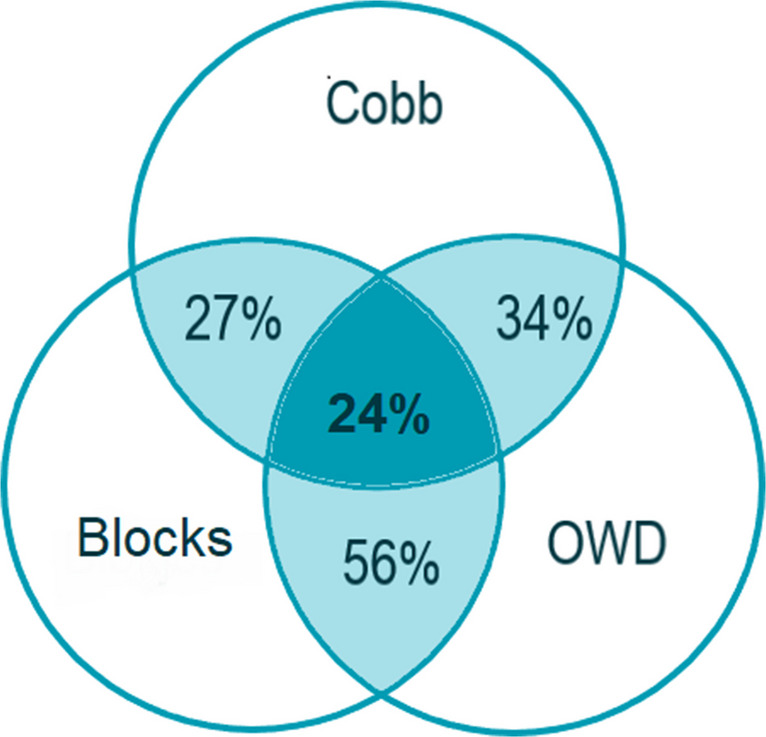
Hyperkyphosis per kyphosis measurement method. Overlap and differences in percentages between the Cobb angle, occiput-to-wall distance (OWD), and blocks method

Base line characteristics are listed in Table 1. The mean age of the 337 participants was 80.0 ± 7.9 years. The hyperkyphosis prevalence varied widely per kyphosis measurement method: 43% measured through the Cobb angle, 65% measured through the blocks method, and 84% measured through the OWD. Only 24% of the participants had hyperkyphosis according to all three measurement methods (Fig. 2).

There were some differences in baseline variables between each group with hyperkyphosis and a normal kyphosis angle, although never consistent in all three analyses. In line with previous literature, more female participants had hyperkyphosis (*p*-values: Cobb 0.293, blocks 0.003, OWD 0.004). Participants with hyperkyphosis also used a higher number of prescription drugs (*p*-values: Cobb 0.261, blocks 0.003, OWD 0.020) and used a walking aid more frequently (*p*-values: Cobb 0.264, blocks 0.022, OWD 0.000). Participants with hyperkyphosis according to the OWD measurement experienced more impairment in ADL tasks (*p*-values: Cobb 0.115, blocks 0.314, OWD 0.006). Remarkably enough, there were no statistically significant differences in the level of physical activity reported by the participants.

Of the 109 participants who performed a TUG, 28% performed the test in 15 s or less. In the AMC hospital, 103 out of the 112 participants included in that study site performed the SPPB. The median score was 7 (IQR 5–9), with only 25% of the participants having a normal score (≥ 9/12 points). The BBS score was normal in 62% of the participants who performed the test (*n* = 65 out of 112 participants). The mean grip strength was 25.0 ± 8.4 kg (*n* = 314). These relatively low mean scores reflect frailty in this study population.

**Table 1:** Baseline characteristics of the total cohort and of hyperkyphotic participants compared to participants with a normal kyphosis angle

	**Overall (n= 337)**	**Hyperkyphosis Cobb**	**Normal kyphosis Cobb**	**Hyperkyphosis blocks**	**Normal kyphosis blocks**	**Hyperkyphosis OWD**	**Normal kyphosis OWD**
**Personal characteristics**	n = 337	n = 122	n = 173	n = 183	n = 146	n = 222	n = 87
Age, mean ± SD (years)	80.0 ± 7.9	80.8 ± 7.2	79.7 ± 8.2	80.7 ± 7.8*	78.9 ± 8.0	80.0 ± 7.9	78.6 ± 8.0
Female (%)	62.9%	65.6%	59.5%	56.3%**	71.9%	57.8%**	75.3%
BMI ([kg/m²], mean ± SD	26.8 ± 5.2	26.2 ± 4.8	27.1 ± 5.7	27.5 ± 5.2*	26.1 ± 5.2	27.2 ± 5.2*	25.8 ± 5.5
Current smoker (%)	16.3%	13.9%	18.0%	14.6%	21.0%	15.0%*	24.7%
Alcohol usage ≥ 1 unit per day (%)	23.4%	24.1%	27.0%	26.7%	23.0%	25.2%	26.3%
**Comorbidities**							
Charlson comorbidity index, median (IQR)	2 (1-3)	2 (1-3)	2 (1-3)	2 (1-3)*	2 (1-3)	2 (1-3)*	2 (1-3)
Cardiovascular comorbidity (%)	82.8%	84.4%	81.5%	85.2%	80.8%	86.0%**	80.4%
MMSE score, median (IQR)	26 (22-28)	26 (23-28)	26 (22-28)	26 (22-28.5)	26 (23-28)	26 (22-28)	26 (23-29)
GDS score, median (IQR)	4 (2-7)	4 (1.5-6.5)	4 (2-7)	4 (2-7)	4 (2-7)	4 (2-7)	3 (1-6)
Total number of drugs, mean ± SD	7.3 ± 4.1	7.0 ± 3.9	7.3 ± 4.1	7.9 ± 4.0**	6.6 ± 4.1	7.6 ± 4.0*	6.4 ± 4.4
**Functional status**							
Use of walking aid (%)	52.8%	55.7%	49.1%	57.9%*	45.2%	58.6%***	34.8%
Impairment ADL-tasks (%)	47.5%	54.1%	44.8%	49.7%	44.1%	51.1%**	34.1%
Impairment IADL-tasks (%)	75.1%	74.6%	75.7%	78.7%*	69.9%	77.6%*	67.4%
**Osteoporosis**							
Osteoporosis in medical history (%)	18.4%	18.9%	19.1%	19.7%	17.1%	18.6%	18.0%
Previous fracture at age >50 years (%)	26.7%	24.6%	28.3%	26.2%	26.7%	29.5%*	16.9%
Vitamin D level [nmol/l], mean ± SD	67.1 ± 34.4	71.1 ± 36.2	66.1 ± 33.1	67.1 ± 34.4	65.7 ± 32.5	68.8 ± 35.2	63.6 ± 32.4

p-value: *** p ≤ 0.001, ** p ≤ 0.01, * p ≤ 0.1

###  Regression analyses

 Table 2 contains the results of the regression analyses. Hyperkyphosis was not associated with  SPPB or hand grip strength. Hyperkyphosis, measured with the blocks method, was  independently associated with a TUG time of ≥15 seconds (adjusted OR 3.86, 95%CI 1.50-9.91, p=0.005). Stratification by sex confirmed the association in women (adjusted OR 3.26, 95%CI 1.26-9.74, p=0.034). In men, the association was not statistically significant (adjusted OR 4.03, 95%CI 0.71-22.75, p=0.114). This was probably due to the lower number of men in these analyses, as the confidence interval is much wider and the direction of the association is similar. Hyperkyphosis, measured with the blocks method, was independently associated with a BBS score of 45 points or lower (adjusted OR 9.42, 95%CI 2.27-39.12, p=0.002). Hyperkyphosis was not associated with the TUG and BBS when measured with the Cobb angle and OWD.

**Table 2:** The association between hyperkyphosis and physical function, results of the regression analyses

	*Cobb*	*Blocks*	OWD
**Grip strength**	*N= 314, B (95% CI)*	*N = 309, B (95% CI)*	*N = 309, B (95% CI)*
Total cohort	0.004 (-0.059-0.067)	-0.302 (-0.624-0.020)	-0.108 (-0.298-0.081)
Females	-0.023 (-0.096-0.050)	-0.111 (-0.504-0.283)	-0.171 (-0.399-0.056)
Males	0.046 (-0.074-0.165)	-0.566 (-1.113—0.018)	-0.019 (-0.350-0.311)
**TUG**	*N = 95, OR (95% CI)*	*N = 105, OR (95% CI)*	*N = 107, OR (95% CI)*
Total cohort	1.09 (0.46-2.60)	3.86 (1.50-9.91) **	1.51 (0.56-4.08)
Females	1.94 (0.47-8.06)	3.26 (1.09-9.72)*	1.07 (0.31-3.72)
Males	0.50 (0.14-1.76)	4.03 (0.71-22.75)	5.99 (0.59-61.11)
**SPPB**	*N = 85, OR (95% CI)*	*N = 100, OR (95% CI)*	*N = 101, OR (95% CI)*
Total cohort	1.39 (0.50-3.93)	1.48 (0.60-3.62)	1.49 (0.50-4.43)
Females	0.53 (0.12-2.42)	3.96 (0.99-15.74)	1.44 (0.35-5.86)
Males	2.40 (0.40-14.58)	0.36 (0.06-2.11)	2.62 (0.30-23.06)
**BBS**	*N = 56, OR (95% CI)*	*N = 64, OR (95% CI)*	*N =65, OR (95% CI)*
Total cohort	1.09 (0.46-2.60)	3.86 (1.50-9.91)**	1.61 (0.59-4.44)
Females	1.94 (0.47-8.06)	3.26 (1.09-9.72)*	1.07 (0.31-3.72)
Males	0.50 (0.14-1.76)	4.03 (0.71-22.75)	5.99 (0.59-61.11)

*p*-value: *** *p* ≤ 0.001, ** *p* ≤ 0.01, * *p* ≤ 0.1. Models adjusted for age, BMI, current or former smoking, alcohol use, fracture after the age of 50and vitamin D level through backward selection.

## Discussion

Hyperkyphosis was highly prevalent and independently associated with the TUG and BBS. Interestingly, competing risks due to comorbidities did not attenuate the association in these older persons with frailty. The association was only present when the kyphosis angle was measured with the blocks method and not with the Cobb angle or OWD. Hyperkyphosis was not associated with the SPPB and hand grip strength.

Although we applied commonly used cut-off scores to define hyperkyphosis, the prevalence of hyperkyphosis differed largely per measurement method. The hyperkyphosis prevalence was particularly high when measured with the OWD. This may underline the large differences between kyphosis measurement methods. The hyperkyphosis prevalence of the Cobb angle is slightly lower than what we expected when compared with other cohorts with physically healthier persons. Conversely, the hyperkyphosis prevalence of the OWD was higher than expected. This might be explained by the relatively low functional level of the participants, resulting in lower back extensor strength and thereby an increase in the kyphosis angle in the standing position.

Congruent with our study results, all studies except the study among the Framingham Heart cohort [9] demonstrated that a larger kyphosis angle was associated with lower physical function on one or more of the tests performed in older adults [1, 7, 10]. The study populations have a higher functional level, and the kyphosis measurement methods and physical function tests used in these studies varied widely, thereby making comparisons between studies and our study difficult. The Framingham study reported no association with gait speed, grip strength, and chair stands time 3.4 years after CT-based Cobb angle measurements. Study results are difficult to compare with ours, as the study population and kyphosis measurement method differ. The study population (*n* = 1100) was much younger than our cohort (mean age 61 ± 8 years versus 80.0 ± 7.9 years), and hyperkyphosis prevalence was low in the subgroup being 65 years and older. Moreover, this subgroup is most likely healthier than our cohort, as mean physical function scores are much higher in the Framingham cohort. This may partly explain the negative results of this study.

Katzman et al. showed cross-sectionally an increase of the TUG of 0.11 s for each standard deviation increase of the kyphosis angle (95% CI 0.02–0.21, *p* = 0.02) [5]. This result is not directly comparable to our study due to the different statistical approach and large differences between this cohort and the participants of our study. The cohort was much younger than our cohort (mean age 68.2 years versus 80.0 years), consisted of only female participants in much better physical condition according to the significantly lower mean scores on the TUG (mean 9.7 ± 2.7 s versus 17 ± 8.5 s). Contrary to our study, Eum et al. showed in female participants that a larger kyphosis angle is associated with a lower SPPB score (B −0.08, 95% CI 0.03–0.21, *p* = 0.01) [6]. This cohort is more similar to our cohort, with a mean age of 79.2 ± 5.6 years and a similar proportion of female participants (63%). Direct comparison of study results is again difficult due to the linear analyses.

Yet, it is noteworthy that even among this older population with frailty and multimorbidity, hyperkyphosis is independently associated with physical function.

Similar to our study, there is a positive association with the TUG in multiple studies, and the association with hand grip strength is less uniform. Although correlation has been shown between grip strength and back extensor strength [39], hand grip strength may be inferior to outcome variables like TUG and SPPB. The association with BBS suggests that balance is an important component of physical function concerning the association with hyperkyphosis. Yet, the SPPB also comprises a balance subtest, and there was no association with the SPPB in the current study, while Eum et al. showed an association with the SPPB. However, as the balance subtest in the SPPB is easier, there may be a ceiling effect in our cohort regarding balance. Three out of four of the participants who performed the SPPB had a high score of 3 or 4 points on this subtest, while only one out of four had a normal score on the SPPB. This may explain why there was an association with the BBS and not with the SPPB in our cohort.

There is also a large variety in kyphosis measurement methods and definitions of hyperkyphosis being used in research. Kyphosis measurements have not been compared directly in these studies, and validation studies are lacking. Concluding which measurement method should be preferred, based only on our cohort study, is impossible. The lack of uniformity underlines the importance of reaching a consensus on the use of kyphosis measurement methods in research. Although this study is not a validation study, we may draw some tentative conclusions.

The blocks method and OWD also incorporate the cervical spine curvature. This curvature increases when the kyphosis angle progresses, potentially contributing to the shift of the center of mass. Therefore, hyperkyphosis measured with the blocks method or OWD may be more indicative of problems with balance, gait, and physical function in general than the Cobb angle. Yet, we found no association between the OWD and physical function in this cohort, which does not support this hypothesis.

The OWD is a measurement method that is easy to conduct in daily clinical practice, yet harder to standardize because of the standing position during the measurement. Especially persons with a very large kyphosis angle are sometimes not able to stand with their heels against the wall. Indeed, the test–retest reliability and the interobserver reliability of the OWD were much smaller than those of the blocks method. This may explain why we found associations with the blocks method and not with the kyphosis measurements in the standing position, i.e., the Cobb angle and OWD. The blocks method is measured in the supine position and therefore leads to a more fixed position, thereby improving the test reliability. Therefore, the blocks method or a Cobb angle measurement in the supine position may result in better test characteristics.

Strengths of this study include the fact that we included a large sample of older persons with frailty, who are highly at risk for physical decline, and the simultaneous measurement of three kyphosis measurement methods. There are two limitations. The first concerns the physical function outcomes. The selection of tests conducted differed partially between the two study centers, and some tests were conducted only if necessary for the diagnostic process. Therefore, a small selection of participants performed the TUG, BBS, and SPPB. This may have resulted in the selection of a subpopulation within this cohort. However, there was no difference in baseline characteristics compared to the total cohort. Furthermore, when choosing a binary logistic approach when linear regression was not possible, the chosen cut-off values may influence study results. We chose the cut-off values for each kyphosis measurement thoughtfully, based on previous literature. Regarding the Cobb angle and blocks method, the cut-off values of this study are commonly used, and the hyperkyphosis prevalence is quite within the expected range based on previously reported prevalences. The prevalence of 84% hyperkyphosis when defining hyperkyphosis with the OWD as 5.0 cm or more is probably too high, based on the previously reported hyperkyphosis prevalence of 55% in a geriatric outpatient clinic. Therefore, the cut-off score most frequently used in literature may be too low for this geriatric outpatient population and may therefore partly explain the negative study results regarding the OWD.

We have chosen the cut-off scores for the TUG, BBS, and SPPB in the regression analyses thoughtfully based on prior research and clinical practice. Yet, studies with normative data, especially for the TUG, show a wide, age-dependent range [40]. So using one value to distinguish between normal and too slow may be too much of a simplification and may disguise differences between age groups. However, as linear regression or using quintiles was not possible due to the distribution, using these cut-off scores in binary logistic regression analysis was, in our opinion, the most suitable solution.

In conclusion, hyperkyphosis was highly prevalent and independently associated with longer TUG times and a lower BBS score in older persons with frailty referred to a geriatric outpatient clinic, only when measured with the blocks method. Hyperkyphosis was not associated with hand grip strength or SPPB. This study adds to the existing literature that even among persons who are highly at risk for functional decline, hyperkyphosis is associated with lower physical function. Potentially, the kyphosis angle is modifiable. Progression of the kyphosis angle can be prevented by treatment of osteoporosis, and reduction of the kyphosis angle has been demonstrated with posture training and back extensor muscle strengthening [41–43]. Early detection and treatment of hyperkyphosis may therefore contribute to the preservation of physical function. Based on the kyphosis measurements in this cohort study, we recommend using the blocks method or Cobb angle in the supine position in future studies to improve comparability and thus the interpretation of study results.The data that support the findings of this study are not openly available due to reasons of sensitivity and are available from the corresponding author upon reasonable request. Data are located in controlled access data storage at the Amsterdam UMC.

## Data Availability

The data that support the findings of this study are not openly available due to reasons of sensitivity and are available from the corresponding author upon reasonable request. Data are located in controlled access data storage at the Amsterdam UMC.
